# Overexpression of mir-135b and mir-210 in mesenchymal stromal cells for the enrichment of extracellular vesicles with angiogenic factors

**DOI:** 10.1371/journal.pone.0272962

**Published:** 2022-08-16

**Authors:** Juliana Maíra Freitas Vieira, Laura Nicoleti Zamproni, Camila H. C. Wendt, Kildare Rocha de Miranda, Rafael Soares Lindoso, Sang Won Han

**Affiliations:** 1 Department of Biophysics, Federal University of São Paulo, São Paulo, Brazil; 2 Department of Biochemistry, Federal University of São Paulo, São Paulo, Brazil; 3 Institute of Biophysics Carlos Chagas Filho, Federal University of Rio de Janeiro, Rio de Janeiro, Brazil; 4 National Center for Structural Biology and Bioimaging/CENABIO, Federal University of Rio de Janeiro, Rio de Janeiro, Brazil; 5 National Institute of Science and Technology for Regenerative Medicine-REGENERA, Federal University of Rio de Janeiro, Rio de Janeiro, Brazil; Università degli Studi della Campania, ITALY

## Abstract

Extracellular vesicles (EVs) are known as molecular carriers involved in cell communication and the regulation of (patho)physiological processes. miRNAs and growth factors are the main contents of EVs which make them a good candidate for the treatment of diseases caused by ischemia, but the low production of EVs by a cell producer and a significant variation of the molecular contents in EVs according to the cell source are the main limitations of their widespread use. Here, we show how to improve the therapeutic properties of mesenchymal stromal cell (MSC)-derived EVs (MSC-EVs) by modifying MSCs to enrich these EVs with specific angiomiRs (miR-135b or miR-210) using lentiviral vectors carrying miR-135b or miR-210. MSCs were obtained from the mouse bone marrow and transduced with a corresponding lentivector to overexpress miR-135b or miR-210. The EVs were then isolated by ultracentrifugation and characterized using a flow cytometer and a nanoparticle tracking analyzer. The levels of 20 genes in the MSCs and 12 microRNAs in both MSCs and EVs were assessed by RT‒qPCR. The proangiogenic activity of EVs was subsequently assessed in human umbilical vein endothelial cells (HUVECs). The results confirmed the overexpression of the respective microRNA in modified MSCs. Moreover, miR-135b overexpression upregulated miR-210-5p and follistatin, whereas the overexpression of miR-210 downregulated miR-221 and upregulated miR-296. The tube formation assay showed that EVs from MSCs overexpressing miR-210-5p (EVmiR210) significantly promoted tubular structure formation in HUVECs. A significant increase in angiogenic proteins (PGF, endothelin 1, and artemin) and genes (VEGF, activin A, and IGFBP1) in HUVECs treated with VEmiR210 justifies the better tubular structure formation of these cells compared with that of EVmiR135b-treated HUVECs, which showed upregulated expression of only artemin. Collectively, our results show that the EV cargo can be modified by lentiviral vectors to enrich specific miRNAs to achieve a specific angiogenic potential.

## 1. Introduction

Peripheral arterial disease (PAD) is a medical condition in which atherosclerotic plaque accumulation leads to narrowing or occlusion of arteries in the lower limbs and thereby a reduced tissue blood supply [1]. In the presence of other comorbidities, such as diabetes, cardiovascular and renal diseases, PAD has higher morbidity and mortality rates [2]. Despite advances in treatments, improvements in long-term outcomes have not been achieved, and noninvasive treatment options have not yet been developed.

Mesenchymal stromal cells (MSCs) have been applied in limb ischemia treatment because these cells can migrate to inflamed ischemic sites by chemotaxis and secrete immunomodulatory and proangiogenic factors that act in a paracrine manner [3–5]. These factors and other molecules released by MSCs can reach the target tissue via extracellular vesicles (EVs), which are natural nanometer-sized membrane structures that mediate cell communication (6). EVs derived from MSCs (MSC-EVs) have shown great therapeutic potential in preclinical and clinical studies and have been described as a novel tool for the treatment of graft-versus-host disease in a case study [6–8]. Furthermore, the observation of the angiogenic capacity in a rat myocardial infarction model after the administration of MSC-EVs showed angiogenic stimulation and reduction of the infarcted area [9]. Similar effects have also been observed in brain and kidney ischemic models [10, 11].

The biological effects mediated by EVs are associated with the direct transfer of mRNAs and microRNAs (miRNAs) in EVs. miRNAs are small noncoding RNAs that control gene expression post transcriptionally and thus participate in several (patho)physiological processes [12]. Among the different regulatory activities of miRNAs, the term “angiomiRs” has been used to describe miRNAs that regulate angiogenesis and promote endothelial cell growth, survival, migration, and their ability to form capillary-like structures [13, 14]. Among angiomiRs, miR-135b is sorted into EVs derived from hypoxia-resistant multiple myeloma cells and enhances angiogenesis through the suppression of hypoxia-inducible factor 1 (HIF-1) [15]. In addition, miR-135b delivered to gastric tumors by EVs negatively regulates forkhead box O1 (FOXO1), a transcription factor associated with angiogenesis regulation, and this regulation results in enhancement of the proliferation, migration, and ring formation of vascular cells [16]. Similarly, miR-210 is another important angiomiR that is upregulated in muscles after acute limb ischemia and thereby promotes angiogenesis and arteriogenesis [17]. In addition, bone marrow-derived MSCs release EVs with more miR-210, which leads to silencing of the antiangiogenic factor EFNA3 and thereby the promotion of angiogenesis by increases in the migration and proliferation of endothelial cells [18].

In this context, it is expected that higher expression of miR-210 or miR-135b could trigger the expression of several angiogenic factors. However, the quality and quantity of these angiogenic factors in MSCs after overexpression of these miRs are unknown, and the content of EVs secreted by these cells is also unclear.

To investigate these questions, we genetically modified MSCs with lentiviral vectors to overexpress miR-135b or miR-210 and then analyzed their gene and miRNA expression profiles. We also assessed the miRNA expression profile and biological activity of MSC-EVs.

## 2. Materials and methods

### 2.1. Animals

All animal experiments were performed only after approval by the Ethics Committee on the Use of Animals of the Federal University of Sao Paulo, Brazil (Approval number: CEUA # 4318140918).

BALB/c mice were obtained from the Center for the Development of Experimental Models in Medicine and Biology of the Federal University of Sao Paulo.

### 2.2. Cells and cell culture

Three cell types were used in this study: MSCs extracted from BALB/c mice and cultivated in DMEM-Low (Dulbecco’s Modified Eagle Medium Low Glucose, Invitrogen, USA) supplemented with 10% heat-inactivated fetal bovine serum (FBS), 2 mM GlutaMAX, and 100 U/mL penicillin‒streptomycin solution; HEK293T cells obtained from the American Type Culture Collection (ATCC) cultivated in DMEM-High (Dulbecco’s Modified Eagle Medium High Glucose, Invitrogen, USA) supplemented with 10% FBS, 2 mM GlutaMAX, and 100 U/mL penicillin‒streptomycin solution; and human umbilical vein endothelial cells (HUVECs, Gibco, Life Technologies, USA) cultured in M200 media with Large Vessel Endothelial Supplement (LVES, GIBCO, USA). All cells were maintained at 37°C in a humidified chamber with 5% CO₂.

### 2.3. Bone marrow MSC isolation and characterization

Bone marrow MSCs were harvested from the femurs, tibia, and humerus of BALB/c mice following a methodology described by Amend et al. [19] and cultivated for three days. The medium was refreshed at intervals of three to four days until adherent cells reached 90% confluence, being defined at this stage as MSCs at passage zero (P0).

For MSC characterization, the capacity of these cells to differentiate into adipocytes and osteoblasts *in vitro* was evaluated using specific differentiation medium for the assessment of adipogenesis or osteogenesis (# A1007001; #A1007201, Gibco Invitrogen, USA), assessment of the expression of the common markers CD73, CD45 (#sc-14682; #sc-25590, Santa Cruz Biotechnology, USA) and CD34 (#ab8158, Abcam, UK) by immunofluorescence and analysis of the expression of CD11b, CD45 (#11-0112-82, #48-0451-82 eBioscience), CD34 (#ab23830 –Abcam, UK), CD73 and CD105 (#127209; #120407—BioLegend, Inc. USA) by flow cytometry (FACSCanto™ II (BD Biosciences) using FACSDiva software (BD Biosciences). The flow cytometry data were analyzed using FlowJo software (FlowJo, OR, USA).

### 2.4. Lentiviral vector construction and production

The customized package plasmid vectors for miR-135b and miR-210 expression (LentimiRa-GFP-mmu-mir-135b - Accession number MI0000646 and LentimiRa-GFP-mmu-mir-210—Accession number MI0000695, ABM Inc., CA, USA) were used to produce lentiviral expression vectors, and their backbone plasmid vector served as a control (pLenti-III-mir-GFP-Blank—ABM Inc., CA, USA). All vectors contained green fluorescence protein (GFP) and puromycin resistance expression cassettes. The plasmid vectors were amplified and purified using the Qiagen mega-prep kit (Sao Paulo, Brazil).

One day before transduction, HEK293T cells were seeded in a 24-well plate (2 x 10^5^ cells/well) and incubated in DMEM-High at 37°C in the presence of 5% CO_2_. For transfection, solutions A and B were prepared. Solution A contained a mix with plasmids, namely, 2 μg of pMDLg/pRRE (Addgene#12251), 1 μg of pRSV-Rev (Addgene#12253), 0.5 μg of pHCMV-VSV-G (n°. AJ315814), and 3 μg of package plasmid (LentimiRa-GFP-mmu-mir-135b, LentimiRa-GFP-mmu-mir-210 or pLenti-III-mir-GFP-Blank) diluted in 180 μL of sterile water and 20 μL of 2 M CaCl_2_ solution. Solution B contained 200 μL of 2X HBS buffer. The solution A was added to the solution B and incubated at room temperature for 15 min, and 100 μL of the solution mixture was then dropped into each well, which contained 2 mL of culture medium. The cells were incubated for 4 h. Subsequently, the medium was replaced with fresh medium, and the cells were incubated until 24 h after transfection. The supernatant with viral particles was collected, centrifuged to remove the remaining cells (5 min at 2000 x g), and stored at –80°C.

To determine viral vector titer, MSCs were seeded in a 24-well plate (1 x 10⁴ cells/well) and each of four viral vector solutions (1, 10, 100, and 300 μL) with 8 μg/mL Polybrene (Sigma, USA) was added in each well. After 72 h of incubation, the cells were fixed with 4% paraformaldehyde and stained with DAPI (1:1000). Images were captured with a fluorescence microscope (Axio Observer Z1, ZEISS, GE), and ImageJ software [20] was used for the counting of GFP-positive cells. The vector titers were calculated using the percentage of GFP-positive cells based on the following formula: (N × P)/V, where N is the number of plated cells, P is the percentage of GFP-positive cells, and V is the volume (mL) of added vector sample.

### 2.5. Construction of MSCmiR, MSCmiR135b and MSCmiR210

For transduction, MSCs were seeded in a 6-well plate (5 x 10^5^ cells/well), and viral particles with 8 μg/mL Polybrene (Sigma, USA) were added. The cells were incubated for 24 h, and the medium was replaced with fresh medium containing 2 μg/mL puromycin (Clontech, USA). Fourteen days after transduction, the cells were maintained in medium containing puromycin, and the medium was refreshed at intervals of three to four days for selection. The efficiency of transduction was assessed based on GFP expression under a fluorescence microscope (Axio Observer Z1, ZEISS, Germany) and double-checked via an immunofluorescence assay with an anti-GFP antibody (Invitrogen, #A-21311) and examination under a confocal microscope (TCS SP8, Leica Microsystems, Germany). The modified cells were named MSCmiRs (cells modified with an empty vector), MSCmiR210s (cells modified to express miR-210), and MSCmiR135bs (cells modified to express miR-135b).

### 2.6. EV isolation and characterization

Prior to EV isolation, MSCs were seeded in T165 bottles (Corning, USA) and incubated until they reached 70–90% confluence. The medium was then discarded, and the cells were washed twice with PBS and cultivated in FBS-deprived medium for 17 to 18 h. The medium collected in this stage was referred to as “conditioned medium”. Differential ultracentrifugation, adapted from Théry et al. [21] was employed to isolate MSC-EVs from the conditioned medium. First, three stepwise centrifugations were performed: 300 x g/10 min, 2000 x g/10 min and 10,000 x g for 30 min. The conditioned medium was stored at -20°C, and two cycles of ultracentrifugation at 100,000 x g for 2 h using a Type 42.1 fixed-angle rotor (Optima XL 100K ultracentrifuge, Beckman-Coulter) were performed with a cleaning step with PBS between them. The pellets were suspended in 100 μL of filtered PBS (0.1 μm, Millipore) and stored at -80°C.

The MSC-EV size distribution and concentration were assessed by nanoparticle tracking analysis (NTA) using NanoSight NS300 (Malvern Panalytical Ltd., Malvern, United Kingdom) and the software provided with the device (NTA 3.4, Malvern, United Kingdom).

The MSC-EV morphology was assessed by transmission electron microscopy (TEM) following a previously described protocol [22]. MSC-EVs were resuspended in a fixative solution containing 2.5% (w/v) glutaraldehyde in 0.1 M cacodylate buffer (pH 7.2). The MSC-EVs were then placed on glow-discharged formvar-coated copper grids with 300 mesh (EMS, Hartfield, PA, USA) for 10 min, and the grids were subsequently negatively stained with 1% (w/v) aurothioglucose (USP, Sigma‒Aldrich) in water for 30 s, dried with filter paper, and examined with an electron microscope operating at 120 kV (Tecnai Spirit microscope, Thermo Fisher Scientific).

For flow cytometry analysis, MSC-EVs (5 x 10^8^ particles) were incubated with 5 μL of 5-μm latex beads (Invitrogen, USA) in a final volume of 100 μL of PBS for 15 min at room temperature with agitation and incubated overnight at 4°C. The exosome markers CD9, CD63, and CD81 (catalog # 124807, 143903, and 104909, respectively, diluted 1:50; BioLegend, USA) were then assessed individually in the EV samples using a flow cytometer (BD FACSAria™ III, BD Biosciences) with FACSDiva software (BD Biosciences). The flow cytometry data were analyzed using FlowJo software (FlowJo, OR, USA).

### 2.7. RNA purification and RT‒qPCR profiling

Total RNA samples were obtained using TRIzol™ reagent (Invitrogen, USA) following the manufacturer’s protocol. RNA quantification was performed using a Nanodrop 2000 (Thermo Scientific), and the RNA purity was evaluated using a spectrophotometer based on a reading absorbance ratio of 260/280. The RNA integrity was assessed by observing intact bands referring to fractions 18S and 28S through the ribosomal RNA bleach agarose gel electrophoresis and using Blue Green Loading Dye (LGC Biotechnology, Brazil) [23]. cDNA was synthesized using the SuperScript IV Reverse Transcriptase kit (Thermo Fisher) for gene quantification and miScript II RT (Qiagen) to determine the miRNA levels.

A panel of 20 genes related to angiogenesis, myogenesis, and fibrogenesis was analyzed to identify potential alterations induced by target miRNA overexpression (S1 Table) [24]. The genes were evaluated by RT‒qPCR using Rotor-Gene Q (Qiagen) and customized RT2 Profiler PCR Array (#CAPM114477, Qiagen) following the protocol described by the manufacturer. In the data analysis, *p* ≤ 0.05 was considered to indicate statistical significance, and fold change (FC) ≥ 2 and FC ≤ 0.5 were considered to indicate positive and negative regulation, respectively.

For miRNA analysis, 12 angiomiRs frequently correlated with angiogenesis and previously appointed as MSC-EV cargo [25] were selected, and their levels in both modified MSCs and MSC-EVs were assessed by RT‒qPCR (S2 Table). The microRNA expression of mature miRNA sequences was evaluated using the Rotor-Gene Q device (Qiagen) and the miScript SYBR Green PCR kit (Qiagen) following the manufacturer’s instructions. miRNAs that satisfied the determined thresholds (*p* ≤ 0.05 and FC ≤ 0.5 or FC ≥ 2) were considered for analysis.

### 2.8. Tube formation assay

Angiogenesis assays were performed using an Angiogenesis Starter Kit (A1460901–Gibco, USA) following the manufacturer’s instructions. In brief, HUVECs were seeded (7.5×10^4^ cells/well) in Geltrex-coated 96-well culture plates, treated with MSC-EVs (2.5 ×10^4^ particles/cell) or PBS (vehicle) and incubated for 10 h at 37°C with 5% CO_2_. Images were captured with an Axio Observer Z1 microscope (Zeiss, Germany) for tube formation analysis at four time points (2, 4, 7 and 10 h). The tubular structures were measured using ImageJ software [20, 26] to evaluate the angiogenic potential of MSC-EVs. For data normalization, the control group (PBS) average was attributed a value of 100%, and measurements of each parameter were compared to the control values. The angiogenic capacity of the MSC-EVs was represented by the mean of five variables (number of extremities, number of branches, number of pieces, number of meshes, and mesh index).

### 2.9. Assessment of angiogenic protein expression in HUVECs after treatment with MSC-EVs

The levels of 55 angiogenesis-related proteins in HUVECs were assessed after treatment with MSC-EVs using a membrane-based sandwich immunoassay (Proteome Profiler Human Angiogenesis Array Kit, R&D systems, USA) following the manufacturer’s protocol. In short, HUVECs were seeded (2×10^5^ cells/well) in 6-well culture plates, treated with MSC-EVs (1x10^8^ particles/mL) and incubated for 24 h at 37°C in the presence of 5% CO_2_. The cells were washed with PBS and lysed with lysis buffer (150 mM NaCl, 5 mM EDTA, 1.0% Triton X-100, 0.1% SDS, 0.5% sodium deoxycholate, and 50 mM Tris-HCl pH 8.0). The cell lysate was centrifuged (13,000 x g, 20 min, 4°C) to remove cell debris, and the protein concentration of the supernatant was then quantified using the Bradford assay (Bio-Rad, USA). Three hundred micrograms of total protein from each sample was mixed with a cocktail of biotinylated detection antibodies following incubation with the array membrane, which was spotted in duplicate with capture antibodies for target proteins. The captured proteins were visualized using a bioluminescence detector (Odyssey® XF, Li-Cor Biosciences, Lincoln, USA) with 8 min of exposure. Image quantification was performed with ImageJ software (version, city, country), and two-way analysis of variance (ANOVA) was used for statistical analysis of the duplicates.

### 2.10. Assessment of angiogenic gene expression in HUVECs after treatment with MSC-EVs

HUVECs were seeded (2×10^5^ cells/well) in 6-well culture plates, treated with MSC-EVs (1×10^8^ particles/mL) and incubated for 24 h at 37°C in the presence of 5% CO_2_. Total RNA was obtained using TRIzol™ reagent (Invitrogen, USA) following the manufacturer’s protocol. The RNA quantity and purity were performed using a Nanodrop 2000. cDNA was synthesized using the High-Capacity cDNA Reverse Transcription Kit. qPCR was performed using Fast SYBR® Green Master Mix and the 7500 Real-time PCR system. All equipment and reagents were obtained from Thermo Fisher Scientific Co. (USA). The primer sequences are shown in S3 Table. For quantification, the target genes were normalized using the geometric mean of the expression of the housekeeping genes beta-actin (*ACTB*) and glyceraldehyde-3-phosphate dehydrogenase (*GAPDH*). The threshold cycles (Ct) were determined for each sample. The relative expression of mRNA was calculated using the 2^(-ΔΔCT)^ method expressed relative to the levels in the nontreated group.

### 2.11. Statistical analysis

The RT‒qPCR data were analyzed using a web tool provided by Qiagen (GeneGlobe Data Analysis Center), in which the *p* value was calculated by Student’s t test of the replicate 2^(-ΔΔCT)^ values for each gene in the control and test group. The data from the tube formation assay were analyzed with GraphPad Prism 9.0 (GraphPad software, EUA) and are presented as the means ± SDs. The treatment groups were evaluated by one-way or two-way analysis of variance (ANOVA) with Bonferroni post hoc test. In all tests, only *p* < 0.05 was considered to indicate a significant difference between groups.

## 3. Results

### 3.1. MSC characterization and miR-135b or miR-210 overexpression

**Fig 1:**
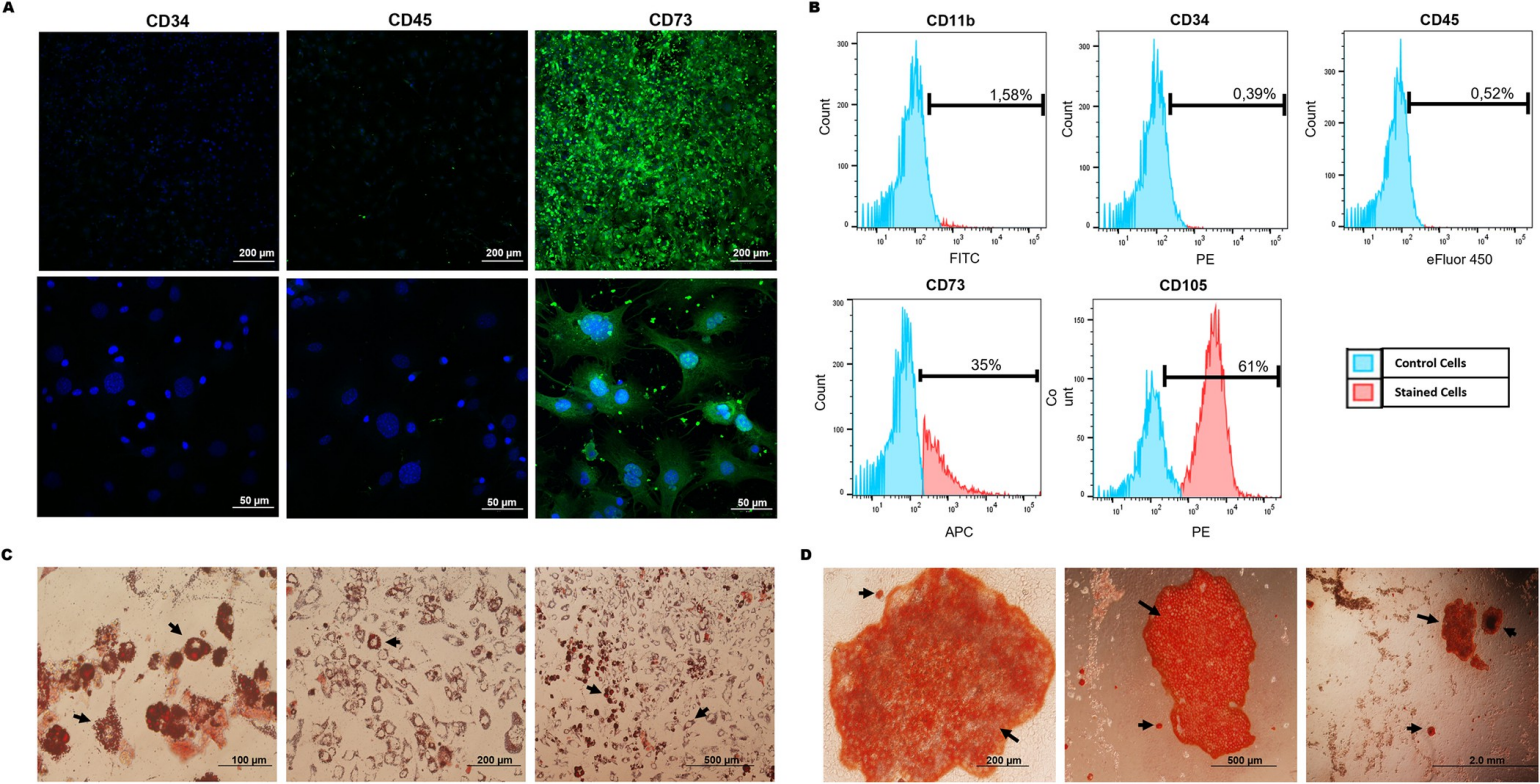
MSC characterization. **(A)** MSC immunofluorescence staining with positive (CD73, in green) and negative (CD34, CD45) markers. The nuclei were stained with DAPI (in blue). **(B)** Flow cytometry phenotyping of MSCs (CD11b, CD34, CD45, CD73, CD105). **(C)** Qualitative assay of adipogenesis. The arrows indicate lipid droplets stained with Oil Red. **(D)** Qualitative assay of osteogenesis. The arrows indicate calcium deposits stained with Alizarin Red.

The characterization of MSCs by immunofluorescence staining and cell cytometry showed that these cells were negative for CD11b, CD34 and CD45 but positive for the classical MSC markers CD73 and CD105 (Fig 1A and 1B). The analysis of the capacity of MSCs to differentiate into adipocytes and osteoblasts showed deposits of lipid droplets and calcium, respectively, confirming the identity of MSCs (Fig 1C and 1D, respectively). To promote overexpression of the angiomiRs, the MSCs were transduced with a lentiviral vector containing a construct for the expression of miR-135b (MSCmiR135bs) or miR-210 (MSCmiR210s).

**Fig 2:**
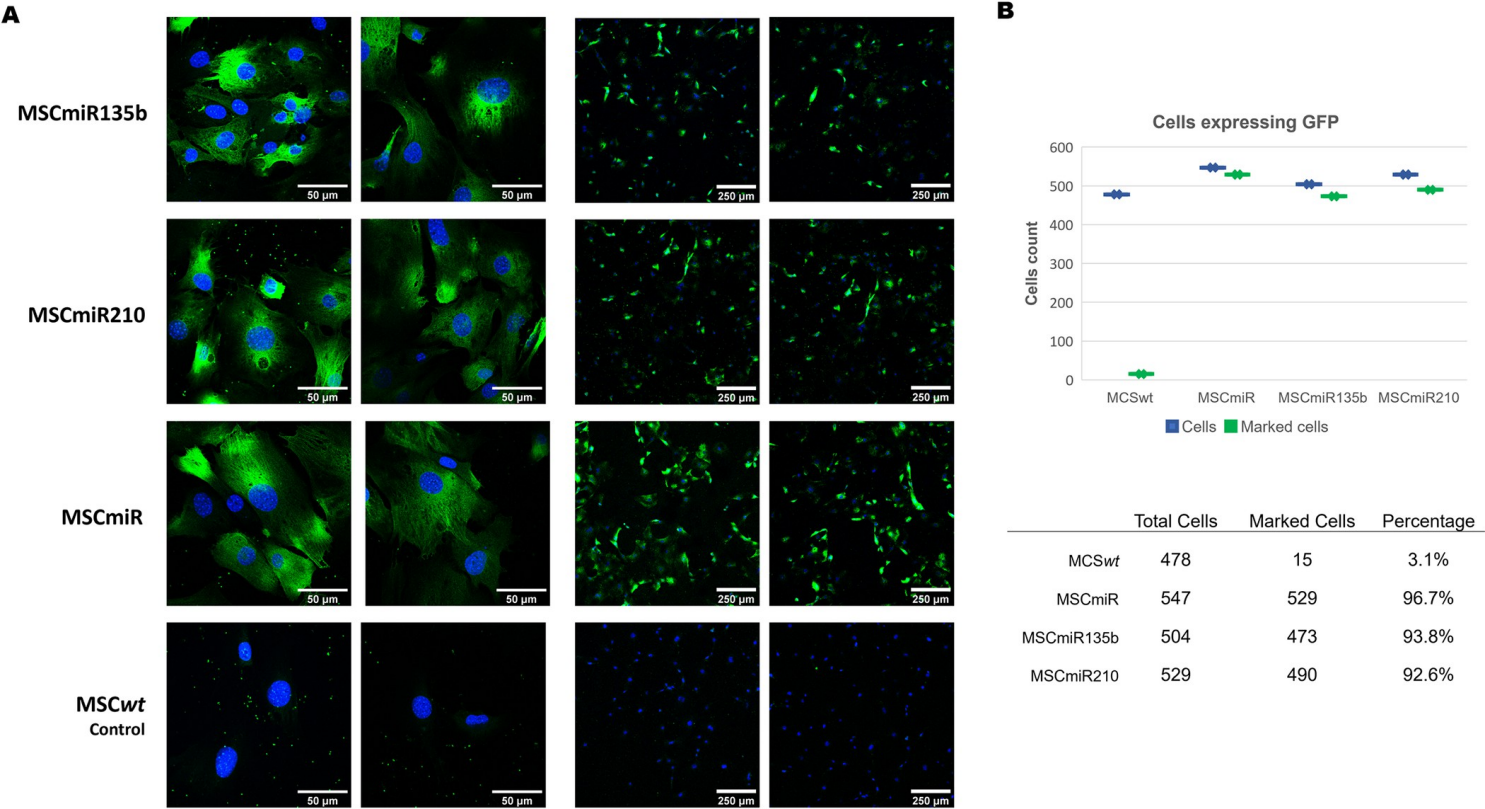
MSCs modified by a lentiviral vector. **(A)** Modified MSCs (MSCmiR135b, MSCmiR210 and MSCmiR) and MSC*wt* (control) in the immunofluorescence assay. The cell nucleus was stained with DAPI (in blue), and GFP was stained with anti-GFP antibody (in green). **(B)** Quantification of MSCs modified with a lentivector by counting of GFP+ cells per mm^2^ of area.

Either miR-135b or miR-210 was successfully transduced into MSCs, as confirmed by immunostaining of GFP+ cells with an anti-GFP antibody, which stained more than 90% of the transduced cells compared with 3.1% of the nontransduced cells (unmodified cells, MSC*wt*s) (Fig 2). MSCs transduced with a vector containing only the miRNA expression construct (MSCmiRs), which were used as a control group, also showed staining for GFP in more than 90% of cells (Fig 2B). Thus, these results show that the MSCs were successfully transduced and selected to express the target miRNAs.

### 3.2. miR-135b overexpression in MSCs promotes follistatin gene expression

To evaluate the effect of miR overexpression in MSCs, we investigated the expression of 20 genes related to angiogenesis by RT‒qPCR. Two control groups were included: MSC*wt* and cells modified with the control vector (MSCmiRs). A comparison of the expression levels between these two groups identified four genes with significant differences in expression (p ≤ 0.05) (Table 1A): *Tgfbr2* (transforming growth factor-β receptor II) was downregulated in MSCmiRs and MSCmiR210s, and *Hgf* (hepatocyte growth factor), *Mstn* (myostatin), and *Fst* (follistatin) were upregulated in MSCmiR135b. To eliminate the interference of the vectors in the analysis, the gene expression levels in these cells were also compared with those in MSCmiRs (Table 1B), which revealed only upregulation of the *Fst* gene in MSCmiR135bs (FC_A_ = 6.2). Because *Fst* is known to encode an angiogenic protein that improves muscle growth by *Mstn* inhibition, the overexpression of miR-135b improves the angiogenic potential of MSCs.

**Table 1:** Gene expression levels in modified MSCs compared with MSC*wt*s and MSCmiR control groups.

		A						B			
		MSC*wt*s						MSCmiRs			
		MSCmiRs		MSCmiR210s		MSCmiR135bs		MSCmiR210s		MSCmiR135bs	
	Gene	FC	*p*	FC	*p*	FC	*p*	FC	*p*	FC	*p*
**Genes with p < 0.05**	*Tgfbr2*	**0.48**	***0*.*0251***	**0.38**	***0*.*0099***	**0.38**	0.6376	0.80	0.5775	**6.83**	0.2942
	*Hgf*	1.68	0.2396	1.35	0.5487	**16.54**	***0*.*0056***	0.84	0.8233	1.19	0.8547
	*Mstn*	**3.69**	0.1486	**4.24**	0.1533	**6.89**	***0*.*0029***	1.15	0.6053	1.87	***0*.*0103***
	*Fst*	**2.37**	0.2276	**3.48**	0.0521	**6.20**	***0*.*0002***	1.47	0.5641	**2.62**	***0*.*0273***
**Genes with p > 0.05**	*Col1a1*	**0.03**	0.222	0.86	0.3827	1.05	0.7531	1.37	0.586	1.67	0.2619
	*Col3a1*	**0.10**	0.2977	**5.68**	0.8723	**5.39**	0.9721	1.54	0.2911	1.47	0.2342
	*Tgfb1*	**0.15**	0.8793	**0.11**	0.483	**0.21**	0.4186	0.99	0.9294	1.20	0.4575
	*Tgfbr1*	1.37	0.4699	**3.61**	0.6591	**7.92**	0.1169	0.83	0.3828	1.82	0.2326
	*Vegfa*	**2.54**	0.2679	**16.51**	0.1622	**6.22**	0.0743	0.98	0.758	**3.18**	0.1723
	*Flt1*	**0.24**	0.1933	**0.46**	0.1568	1.36	0.7677	**0.47**	0.1997	1.39	0.6453
	*Kdr*	0.79	0.7193	1.49	0.6651	**2.54**	0.6596	0.83	0.7696	1.40	0.4070
	*Fgf1*	0.82	0.8749	1.55	0.6397	1.84	0.8733	1.15	0.7426	1.36	0.7871
	*Hif1a*	**9.80**	0.1614	**10.70**	0.0829	1.68	0.4878	1.09	0.9046	1.27	0.4377
	*Csf2*	0.60	0.4123	0.64	0.3738	0.72	0.7760	0.95	0.9275	1.01	0.5531
	*Csf2ra*	0.97	0.8978	0.70	0.4040	**2.73**	0.6845	1.03	0.9274	1.12	0.9829
	*Csf2rb*	0.92	0.9076	**2.65**	0.6239	**3.03**	0.3561	1.17	0.9485	1.34	0.5676
	*Pax3*	1.22	0.7260	1.51	0.6750	1.10	0.6617	1.24	0.9362	0.90	0.4790
	*Pax7*	**0.22**	0.4246	1.93	0.9491	**2.01**	0.8706	0.82	0.6201	0.85	0.3430
	*Myod1*	0.58	0.5449	0.96	0.8288	1.98	0.1660	0.76	0.3322	1.57	0.4702
	*Mtor*	1.08	0.7332	1.00	0.8330	1.63	0.2078	0.93	0.6207	1.51	0.2980

The RT‒qPCR data were obtained from four samples of each group and normalized to the housekeeping gene *GAPDH*. Positive regulation (FC ≥ 2) and negative regulation (FC ≤ 0.5) are shown in bold font. The values with significant p values (*p* ≤ 0.05) are shown in italicized bold.

### 3.3. Target miRNAs are successfully upregulated in modified MSCs

To explore the impact of target miRNA expression in the MSC-EV cargo, the expression of 12 angiomiRs in the modified MSCs was assessed by RT‒qPCR. First, we assessed the effect of lentiviral transduction in MSCs by comparing the expression levels between MSCmiRs and MSC*wt*s (Table 2A). A *p* value lower than 0.05 indicates that the expression of the indicated miRNA was affected by lentiviral transduction, independent of target miRNA overexpression. Thus, the FC values of the indicated miRNA need to be cautiously used to determine the expression levels.

**Table 2:** miRNA expression in modified MSCs compared with that in MSC*wt*s and MSCmiRs (control cells).

		A									B					
		MSC*wt*s									MSCmiRs					
		MSCmiRs			MSCmiR210s			MSCmiR135bs			MSCmiR210s			MSCmiR135bs		
	miRNA	FC	*p*	#	FC	*p*	#	FC	*p*	#	FC	*p*	#	FC	*p*	#
Anti-angiomiRs	miR-15a-5p	1.47	0.359	*** ***	1.31	0.478	*** ***	1.32	0.483	*** ***	0.89	0.634	*** ***	0.90	0.940	
	miR-34a-5p	0.63	0.680	*** ***	**0.40**	0.073	*** ***	**0.24**	0.202	A	0.63	0.380	*** ***	**0.38**	0.550	A
	miR-221-3p	1.86	***0*.*013***	*** ***	0.95	0.904	*** ***	**3.00**	0.105	*** ***	0.51	***0*.*017***	*** ***	1.61	0.232	
	miR-222-3p	1.41	0.072	*** ***	0.89	0.734	*** ***	1.96	0.203	*** ***	0.63	0.073	*** ***	1.39	0.274	
Pro-angiomiRs	miR-21-5p	0.95	0.672	*** ***	1.18	0.957	*** ***	1.15	0.652	*** ***	1.25	0.384	*** ***	1.21	0.400	
	miR-126-3p	1.44	0.483	*** ***	0.95	0.716	*** ***	**0.07**	***0*.*041***	A	0.66	0.441	*** ***	**0.05**	0.261	A
	miR-130a-3p	0.83	0.434	*** ***	1.00	0.869	*** ***	1.10	0.626	*** ***	1.21	0.170	*** ***	1.32	0.343	
	miR-296-5p	1.15	0.557	*** ***	1.56	0.052	*** ***	1.97	0.177	*** ***	1.35	***0*.*005***	*** ***	1.71	0.203	
Experimental AngiomiRs	miR-135b-5p	**4.33**	0.266	B	**2.24**	0.250	B	**569.41**	0.052	A	0.52	0.482	B	**131.60**	0.053	A
	miR-135b-3p	1.55	0.996	B	0.68	0.541	B	**95.23**	0.055	B	**0.44**	0.289	B	**61.39**	0.054	B
	miR-210-5p	1.59	***0*.*035***	*** ***	**2.12**	***0*.*005***	*** ***	**3.29**	***0*.*027***	*** ***	1.33	0.067	*** ***	**2.07**	0.062	
	miR-210-3p	1.78	***0*.*019***	*** ***	**4.43**	***0*.*005***	*** ***	**2.98**	0.135	*** ***	**2.49**	***0*.*011***	*** ***	1.67	0.277	

The RT‒qPCR data were obtained from three samples in duplicate from each group and normalized to the housekeeping gene *RNU6*. Positive regulation (FC ≥ 2) and negative regulation (FC ≤ 0.5) are shown in bold font. Values with significant p values (p ≤ 0.05) are shown in italicized bold font. Values close to the threshold are underlined. # Observation → A: Ct value is relatively high (> 33) in one of the compared samples (control or test) and low in the other; B: the Ct of the corresponding gene is relatively high (Ct > 33).

The miRNA expression of the modified cells (MSCmiR210s and MSCmiR135bs) was then compared with that in the MSCmiRs (Table 2B), and *p* ≤ 0.05 was considered significant. Here, we also included groups with a p value close to 0.05 for discussion. Based on these criteria, we found three miRNAs that were altered in MSCmiR210s: miR-210-3p (FC = 2.49; p = 0.011), miR-210-5p (FC = 1.33; p = 0.067) and miR-296-5p (FC = 1.35; p = 0.005). Although the FCs of miR-210-5p and miR-296-5p were below the FC threshold and the p value of miR-210-5p was slightly higher than 0.05, an analysis of the p values of these miRNAs in MSCmiRs, which indicates the influence of lentiviral vector transduction on miRNA expression, indicates that only miR-296-5p (p = 0.557) exhibited a significant FC increase.

Similarly, three altered miRNAs were observed in MSCmiR-135bs: miR-210-5p (FC = 2.07; p = 0.062), miR-135b-5p (FC = 131.6; p = 0.053) and miR-135b-3p (FC = 61.39; p = 0.054). These three miRNAs had a FC higher than 2, as indicated in both Table 2A and 2B, and their p values were close to 0.05, indicating a tendency of upregulation. In particular, in MSC*wt*s, the two forms of miR-135b had high Ct values (>33) in the qPCR analysis, suggesting that this miRNA has marginal expression in unmodified cells, resulting in the high FC values shown in Table 2. However, this assumption needs to be confirmed by absolute quantification through qPCR. Overall, these results confirm the successful overexpression of the target miRNA in MSCs and demonstrate that their overexpression may also lead to changes in other pro- and anti-angiomiRs.

### 3.4. MSC-EV characterization

**Fig 3:**
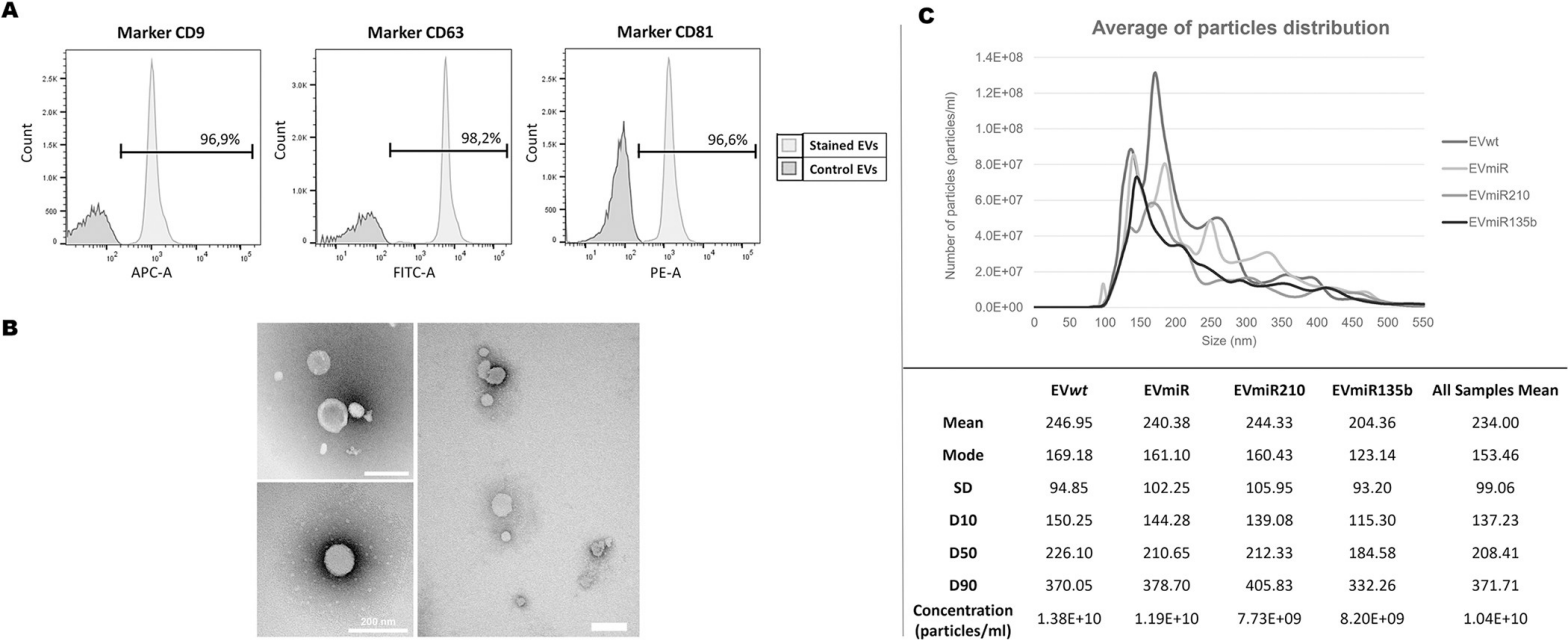
MSC-EV characterization. **(A)** Surface markers CD9, CD63 and CD81 of EV*wt*s detected by flow cytometry. **(B)** Representative TEM images of EV*wt*s with a rounded or elliptical morphology. **(C).** Size and particle distribution (concentration) obtained by NTA of EVs from transduced and nontransduced cells. The plots represent the average from 4 samples per group, and five recordings of each sample were performed. EV*wt*s = MSC*wt*-derived EVs; EVmiRs = MSCmiR-derived EVs; EVmiR210s = MSCmiR210-derived EVs; EVmiR135bs = MSCmiR135b-derived EVs.

MSC-EVs were characterized by three methods: NTA (size and distribution), TEM (size and morphology), and flow cytometry (presence of common membrane surface markers). Flow cytometry analysis revealed that the MSC-EVs exhibited more than 96% positive staining for EVs based on the three typical exosome tetraspanins (CD9, CD63, and CD81) (Fig 3A).

Furthermore, the TEM images showed the distinctive ellipse or rounded morphology and heterogeneous size distribution of EVs (Fig 3B). The size distribution results were supported by the NTA analyses (Fig 3C), which revealed that the MSC-EV samples appeared to be a mixture of exosomes and MVs that mainly ranged from 130 nm to 370 nm, with a mode of approximately 153 nm. This type of mixed EV sample is a typical result obtained by the chosen method for production and purification.

Additionally, the results suggest that transduction with lentivectors did not change the EV profiles because the samples had similar size and distribution values with little divergence, possibly due to experimental variation. Collectively, the results from the analyses of the morphology, size distribution, and membrane markers indicate that the sample was indeed an EV population enriched in exosomes.

### 3.5. Modified MSCs secrete EVs with different microRNA signatures

The biological activity of EVs is highly dependent on their cargo. Therefore, the variations in miRNAs in MSC-EVs were analyzed by RT‒qPCR. The miRNA levels in EVs were analyzed following the above-described steps, and the values were compared with two control groups: EVs derived from MSCmiR (EVmiRs) and EVs derived from MSC*wt* (EV*wt*s). The results show an overall reduction in the expression of most investigated miRNAs. The exceptions were miR-15a-5p in all the groups, miR-135b-5p in EVmiR135bs (FC = 172.45), and miR-210-3p, miR-135b-3p and miR-135b-5p in EVmiR210s (FC = 1.58, 13.77 and 7.48, respectively).

However, the comparison of EVmiRs and EV*wt*s (Table 3A) indicated that the miR-15a-5p levels were affected by lentiviral transduction and that the miR-135b FC values of EVmiRs were minimal, with *p* > 0.05, and this miRNA should thus be disregarded. In contrast, the miR-210-5p levels were negatively affected by lentiviral transduction. Thus, despite the downregulation shown in Table 3A, cellular miRNA upregulation led to an increase in the miR-210-5p levels in the EVmiR210 cargo. In contrast, in EVmiR135bs, a significant increase in the miR-135b-5p levels were detected in both comparisons (Table 3A and 3B). Altogether, these data indicate that EVs derived from modified MSCs were enriched with the target miR and exhibited low levels of other miRNAs.

**Table 3:** miRNA expression in MSC-EVs compared with that in EV*wt*s and EVmiRs (control groups).

		A									B						
		EV*wt*s									EVmiRs						
		EVmiRs			EVmiR210s			EVmiR135bs			EVmiR210s				EVmiR135bs		
	miRNA	FC	*p*	#	FC	*p*	#	FC	*p*	#	FC	*p*	#	FC		*p*	#
Anti-angiomiRs	miR-15a-5p	**7.85**	0.252	B	**15.00**	***0*.*000***	*** ***	**19.61**	0.357	B	1.91	0.922	B	**2.50**		0.420	B
	miR-34a-5p	**0.41**	0.337	B	**0.13**	0.261	B	**0.06**	0.246	B	**0.32**	0.088	B	**0.15**		0.051	B
	miR-221-3p	**0.37**	0.231	*** ***	**0.30**	0.196	*** ***	**0.27**	0.186	*** ***	**0.36**	***0*.*034***	*** ***	0.56		0.127	
	miR-222-3p	0.79	0.512	A	**0.28**	0.287	A	**0.44**	0.347	A	0.84	0.402	*** ***	0.80		0.274	
Pro-angiomiRs	miR-21-5p	0.86	0.548	*** ***	0.58	0.381	*** ***	**0.36**	0.286	*** ***	0.67	***0*.*001***	*** ***	**0.41**		***0*.*001***	
	miR-126-3p	**0.36**	0.272	A	**0.22**	0.230	B	**0.47**	0.330	B	0.63	0.195	A	1.33		0.247	A
	miR-130a-3p	0.54	0.221	B	0.50	0.220	B	0.73	0.263	B	0.94	0.969	B	1.36		0.484	B
	miR-296-5p	1.28	0.441	A	**0.31**	0.378	B	0.62	0.399	B	**0.24**	***0*.*041***	A	**0.49**		0.169	A
Experimental AngiomiRs	miR-135b-5p	**0.09**	0.220	C	0.68	0.438	B	**15.60**	***0*.*028***	*** ***	**7.48**	***0*.*000***	*** ***	**172.45**		***0*.*019***	
	miR-135b-3p	**0.09**	0.220	C	1.25	0.789	B	**0.47**	0.718	B	**13.77**	***0*.*000***	*** ***	**5.17**		0.324	B
	miR-210-5p	**0.08**	***0*.*002***	*** ***	**0.13**	***0*.*003***	*** ***	**0.05**	***0*.*002***	*** ***	1.58	***0*.*046***	*** ***	0.64		0.240	B
	miR-210-3p	**0.37**	0.231	*** ***	**0.30**	0.196	*** ***	**0.27**	0.186	*** ***	0.81	0.341	*** ***	0.75		0.208	

The RT‒qPCR data were obtained from three samples in duplicate for each group and normalized to the housekeeping gene *RNU6*. Positive regulation (FC ≥ 2) and negative regulation (FC ≤ 0.5) are shown in bold font. Values with significant p values (p ≤ 0.05) are shown in italicized bold font. # = Observation → A: the Ct value is relatively high (> 33) in one of the compared samples (control or test) and low in the other; B: the Ct of the corresponding gene is relatively high (Ct > 33); C: the mean Ct of the corresponding gene was not determined or is greater than the defined cutoff (Ct > 40).

### 3.6. EVs enriched with miRNA 210 improve angiogenesis

**Fig 4:**
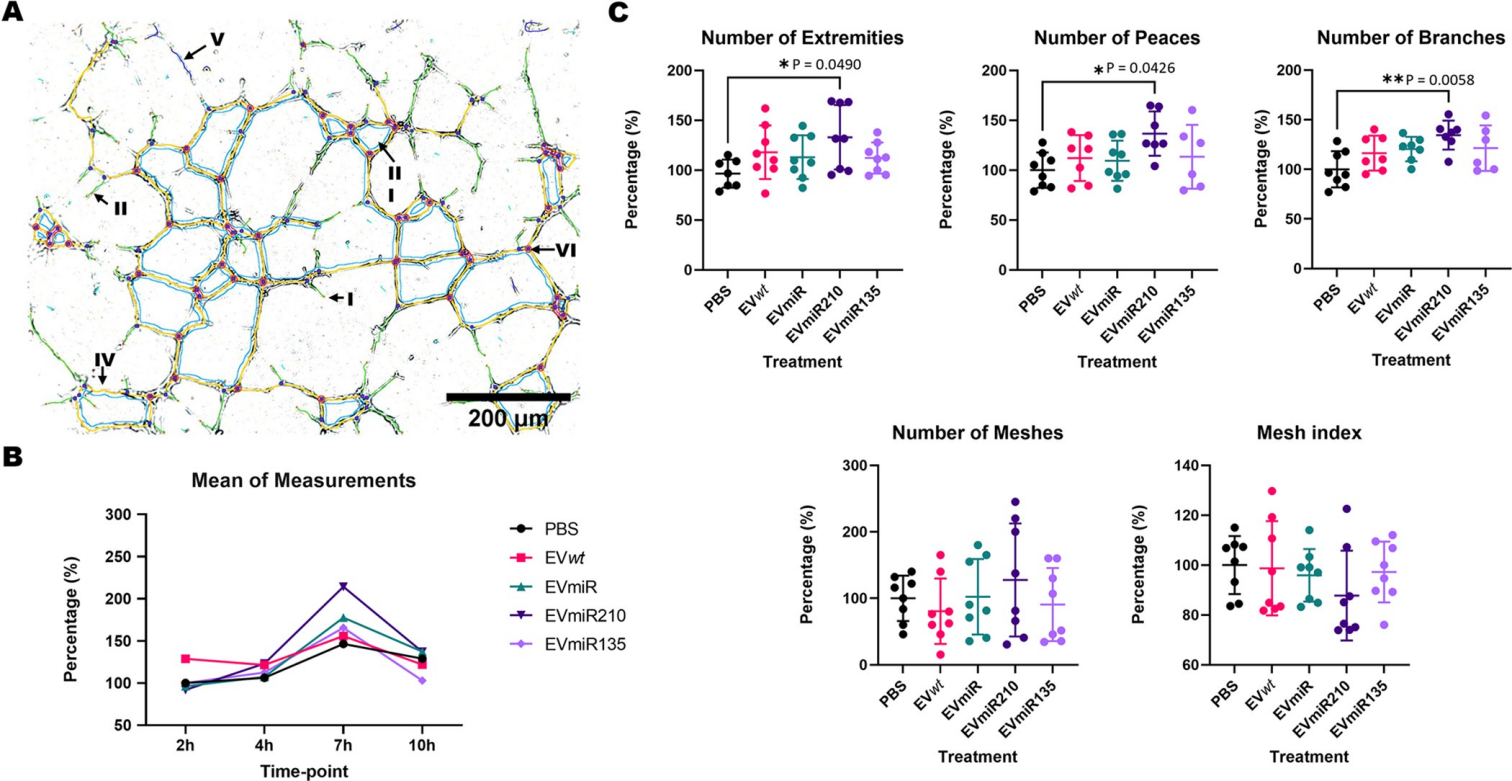
Tube formation assay with MSC-EVs and HUVECs. **(A)** Representation of the investigated tubular structures, which were highlighted by Angiogenesis Analyzer in ImageJ software: extremities (I–red), branches (II–green), meshes (III–light blue), segments (IV–yellow), isolated segments (V–navy blue), and junctions (VI–pink circle). **(B)** Mean measurements of the tubular structures from HUVECs treated with PBS or MSC-EVs at 4 time points (2, 4, 7 and 10 h). **(C)** Analysis of five types of tubular structures in HUVECs treated with PBS or MSC-EVs at the 7-h time point. EV*wt*s = MSC*wt*-derived EVs; EVmiRs = MSCmiR-derived EVs; EVmiR210s = MSCmiR210-derived EVs; EVmiR135bs = MSCmiR135b-derived EVs. The significance of the differences were assessed by one-way ANOVA and Bonferroni correction for the post hoc analysis.

Angiogenesis can alleviate ischemic damage and thus reduce fibrosis and cell death in the affected tissue. To test whether the changes in the MSC-EV cargo affected the biological effects of these EVs, HUVECs were incubated with MSC-EVs, and the resulting angiogenic properties at different time points were assessed by tube formation assays (Fig 4A and 4B). Measurements were made at 7 h, which was the time point with the higher amount of tubular structures.

**Fig 5:**
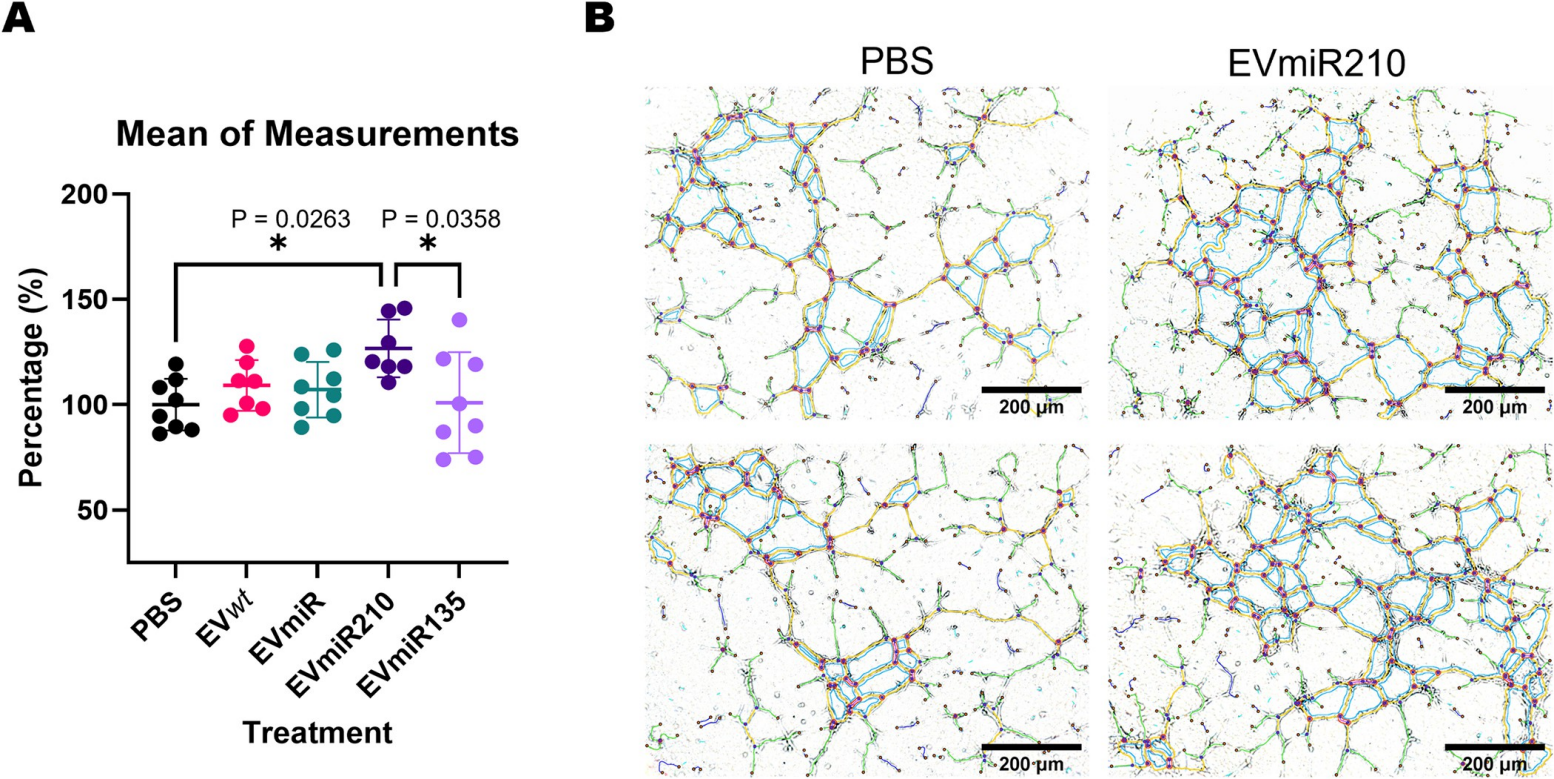
Angiogenic activity of EVmiR210 in endothelial cells. **(A)** Mean of the five structures analyzed in HUVECs treated with PBS or MSC-EVs at the 7-h time point. **(B)** Comparative image of HUVECs treated with PBS or EVmiR210 with tubular structures, which are highlighted by Angiogenesis Analyzer in ImageJ software. EV*wt*s = MSC*wt*-derived EVs; EVmiRs = MSCmiR-derived EVs; EVmiR210s = MSCmiR210-derived EVs; EVmiR135bs = MSCmiR135b-derived EVs. The significance of the differences was assessed by one-way ANOVA and Bonferroni correction for the post hoc analysis.

The results showed a significant increase in 3 of 5 parameters in HUVECs treated with EVmiR210 compared with the levels found for the PBS control group (Fig 4C). Similarly, the mean of the measurements obtained for HUVECs incubated with EVmiR210 was significantly increased by approximately 27% (*p* = 0.026) in comparison with the values obtained for the PBS control group (Fig 5). Similar results were not found for the other MSC-EVs (EV*wt*s, EVmiRs, and EVmiR135bs), indicating a singular angiogenic property of EVmiR21.

### 3.7. EV-miR210 increases the angiogenesis-related protein levels in HUVECs treated with MSC-EVs

**Fig 6:**
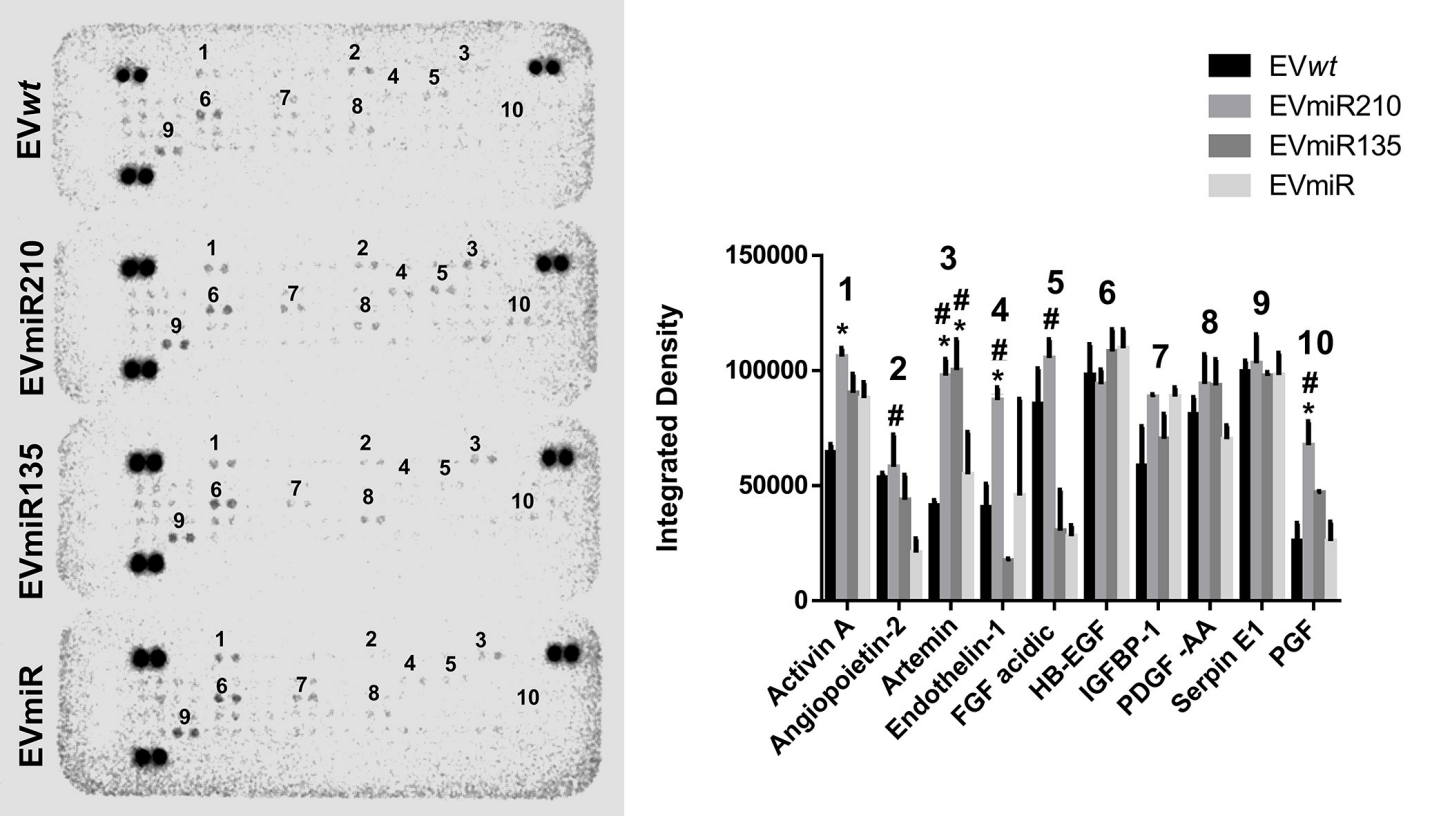
Profile of angiogenic-related proteins in HUVECs after treatment with EVmiRs. The membrane was exposed for 8 min in a bioluminescence detector (left), and the intensity of each spot was measured using ImageJ software (right). The significance of the differences was analyzed by two-way ANOVA and Bonferroni correction for the post hoc analysis. * indicates p < 0.05 in comparison with EV*wt*s; # indicates p < 0.05 in comparison with EVmiRs. EV*wt*s = MSCwt-derived EVs; EVmiRs = MSCmiR-derived EVs; EVmiR210s = MSCmiR210-derived EVs; EVmiR135bs = MSCmiR135b-derived EVs.

In the membranes incubated with the cell extracts from HUVECs treated with EVmiR210 or EVmiR135b, 10 growth factors were noticeably marked among the 55 factors in the panel: activin A, angiopoietin 2, artemin, endothelin-1, acidic fibroblast growth factor, heparin-binding epidermal growth factor, insulin-like growth factor-binding protein, platelet-derived growth factor-AA, serpin-1 and placental growth factor (Fig 6). However, after eliminating the effects in the control groups, EV*wt*s and EVmiRs, only artemin, endothelin-1 and PGF were upregulated after treatment with EVmiR210, and only artemin was upregulated after treatment with EVmiR135b.

### 3.8. EVmiR210 increases angiogenic gene relative expression in HUVECs treated with MSC-EVs

**Fig 7:**
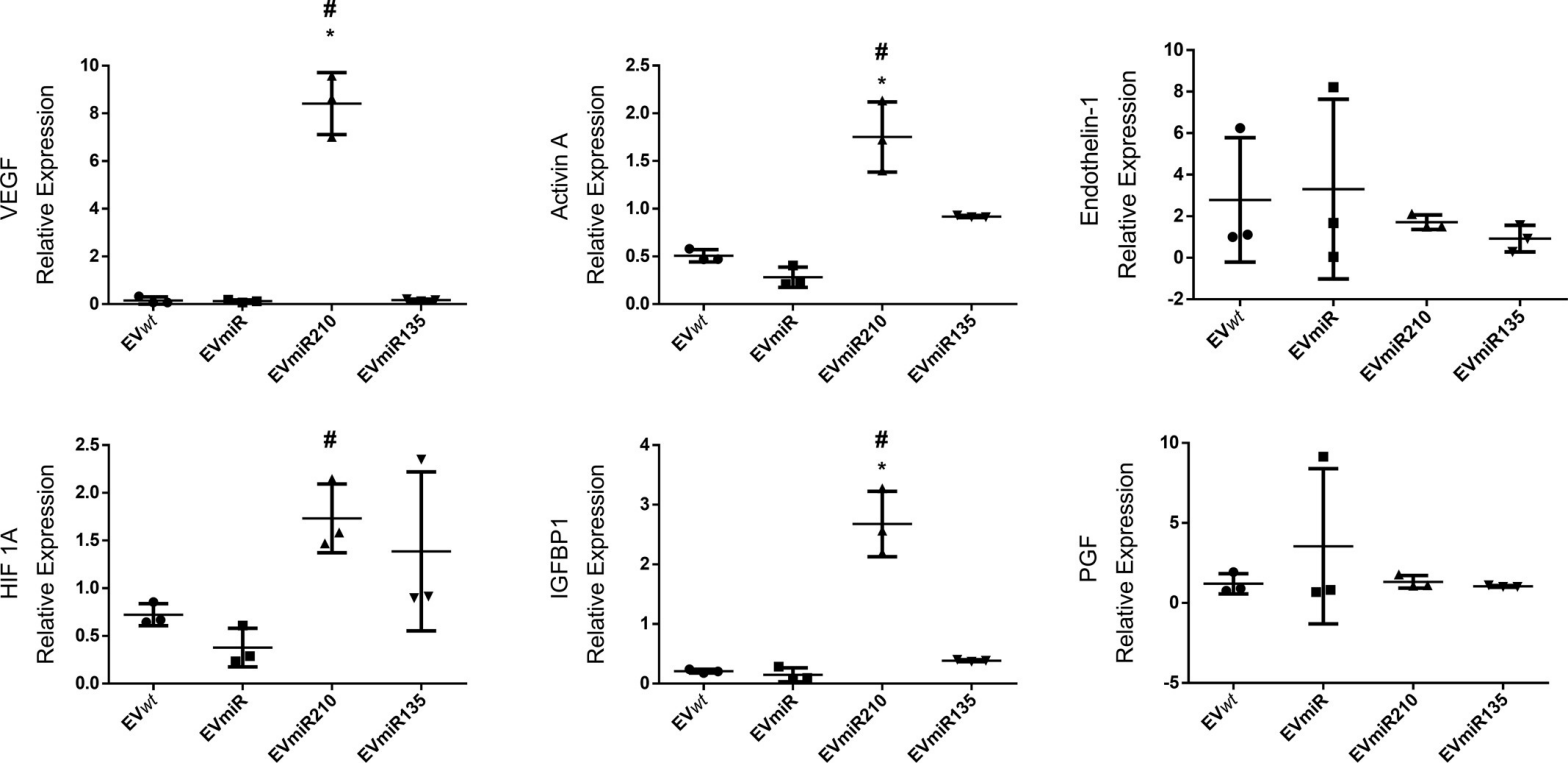
Angiogenic gene expression profile of HUVECs after treatment with EVmiRs. Relative gene expression was determined as described in the Materials and Methods section. The significance of the differences was analyzed by one-way ANOVA and Bonferroni correction for the post hoc analysis. * indicates p < 0.05 in comparison with EV*wt*s; # indicates p < 0.05 in comparison with EVmiRs. EV*wt*s = MSCwt-derived EVs; EVmiRs = MSCmiR-derived EVs; EVmiR210s = MSCmiR210-derived EVs; EVmiR135b = MSCmiR135b-derived EVs.

After identification of the overexpressed angiogenic proteins in HUVECs after treatment with EVmiRs, the 4 genes (PGF, IGFBP1, ET-1 and INHBA) identified in this study and 2 classical angiogenic genes (HIF-1A and VEGF) were assessed by RT‒qPCR (Fig 7). In this panel, VEGF, INHBA and IGFBP1 were upregulated after treatment with EVmiR210 but not after treatment with EVmiR135b. HIF-1A was upregulated in both EVmiR210s and EVmiR135bs, but the p values for the comparison with the EVmiRs and EV*wt*s were higher than 0.05.

## 4. Discussion

The importance of EVs and miRNAs in biological functions is well accepted today, but several challenges remain in the production of a specific miRNA-EV system for therapy. Here, we demonstrated that the overexpression of miR-135b or miR-210 in MSCs by a lentiviral vector can alter the mRNA and miRNA levels in the secreted EVs and thus improves their angiogenic potential without altering their secretion rate or morphology.

MSCs from the bone marrow and adipose tissue are the main sources for clinical cell therapy and EV production [27, 28]. Modification of these cells with lentiviral vectors is well documented for exogenous gene expression and gene therapy [29, 30]. Collectively, EV production with a specific miRNA via MSCs modified using a lentiviral vector represents an interesting strategy for producing specific miRNA-loaded EVs for precise targeted therapy. Additionally, the overexpression of miRNAs in EVs reduces the need for large amounts of EVs to achieve the same beneficial effect, which results in reductions in both cost and time and thus facilitates translation to the clinic.

*Fst* was positively regulated in MSCs overexpressing miR-135b. This gene regulates the activity of activin A, an important protein in inflammation. *Fst* might be upregulated in MSCmiR135bs because activin A receptor type-1 is one of the predicted targets of miR-135b using the public mouse database TargetScan (version 7.1, http://www.targetscan.org). *In vitro* studies have shown that *Fst* associated with VEGF can facilitate new blood vessel formation in tumors [31, 32]. Furthermore, in myogenesis, *Fst* plays an important role as a myostatin antagonist and is the main inhibitor of muscle growth [33]. The administration of follistatin exerts a relevant therapeutic effect on spinal muscular atrophy in an animal model [34]. Hence, increased *Fst* expression may represent a gene regulation that is favorable for angiogenesis and myogenesis, which is an essential physiological need for the recovery of ischemic and atrophic skeletal muscle.

An analysis of the miRNA levels revealed that MSCmiR210 upregulated two forms of miR-210 (miR-210-5p and miR-210-3p). MSCmiR135bs showed increased expression of both miR-135b forms (miR-135b-5p and miR-135b-3p). These data indicate the desired increase of miRNA target expression, particularly the expression of miR-135b, which stands out with a FC higher than 60-fold (this result is further discussed in this section). The MSCmiR135bs also exhibited upregulation of miR-210-5p. A relationship between miR-210 and miR-135 is expected because both are implicated in the HIF-1 pathway, and higher levels of these two miRNAs may indicate the production of more angiogenic factors by these cells [35].

Furthermore, MSCmiR210s also showed a slight increase in miR-296 expression. This miRNA inhibits tyrosine kinase substrate expression regulated by hepatocyte growth factor (HGF). This inhibition leads to higher expression of VEGFR2 (VEGF receptor 2) and PDGFR-β (platelet-derived growth factor-beta receptor), resulting in the promotion of angiogenesis [36]. In addition, miR-221 was downregulated in MSCmiR210s. miR-221 is involved in vascular remodeling, silencing the c-KIT receptor, and decreasing the level of VEGFR2 expression [37]. Thus, miR-296 and miR-221 regulate the VEGF pathway, similar to miR-210, and in this study, their variation was correlated with a higher level of VEGF expression, which stimulates a favorable environment for angiogenesis. Therefore, the MSC data show that the overexpression of miR-135b or miR-210 in MSCs promotes the expression of genes and miRNAs related to angiogenesis and myogenesis, which are beneficial for the treatment of ischemic limbs.

One of our concerns in the modification of MSCs with a lentiviral vector is the possibility of altering EV properties, such as the size and membrane proteins, because these alterations can affect the biological activity of EV and thereby EV-based therapy. The isolated EVs displayed a distinctive morphology (Fig 2B) and a size distribution predominantly under 250 nm (Fig 2C), which are typical characteristics of EVs [38, 39]. In addition, all three exosome tetraspanin markers were present on EVs (Fig 2A), which showed that the modification of MSCs with lentiviral vectors does not affect the characteristics of EVs.

In contrast, the EV cargo exhibited different levels of the investigated miRNAs. Although the EVmiR135b cargo showed an increase in miR-135b-5p expression, the EVmiR210s had higher levels of miR-210-5p, although this increase was less evident. These results indicate that the “5p” isomiR for both miRNAs is the dominant form [40], which leads to the hypothesis that this isomiR may have more influence on cell communication than the “3p” isomiR. Furthermore, this result also demonstrates that EVs are enriched with the overexpressed miRNAs, confirming our early speculation.

An angiogenesis assay revealed that the EVmiR210 group promoted better tubular structure formation. Hence, the angiogenic potential of this group was superior to that of EVmiR135bs, which showed no significant difference in comparison to the control groups. A significant increase in angiogenic proteins (PGF, endothelin 1 and artemin) and genes (VEGF, activin A and IGFBP1) in HUVECs treated with VEmiR210 justifies the better tubular structure formation of these cells compared with that of EVmiR135b-treated HUVECs, which showed upregulated expression of only artemin. Nevertheless, although we used the same amount of EVs, the absolute concentration of their contents is unknown; thus, caution should be taken with this interpretation because the biological activity depends on the miRNA concentration. Barter et al. [41] showed that the expression of miR-135b was markedly lower than that of miR-210 in human bone marrow MSCs. This information corroborates our hypothesis that the FC values found in MSCmiR135bs and EVmiR135bs are likely due to the low expression of miR135b in MSCs. This observation also raises suspicion that the amount of miR-135b in EVs could be lower than that of miR-210, which would promote fewer tubular structures. Therefore, further absolute quantification of these miRNAs by real-time PCR should clarify the above discussion. Moreover, the changes in other miRNAs in EVs were not investigated, which limits the understanding of the miRNA molecular signature of EVs and their regulatory mechanism.

Collectively, our results show that EVs enriched with a specific miRNA can be produced by MSCs modified with lentiviral vectors expressing the target miRNA. In addition, we found that EVmiR210 improved the angiogenic activity. These results are relevant for the application of specific miR-enriched EVs in regenerative medicine by improving our understanding of EV production as well as the regenerative and repair processes of EVs (9,46). Moreover, despite these in vitro results under normoxia, the true potential of EVmiR210s for the treatment of limb ischemia still needs to be investigated in an animal model, and this promising result opens a window to be explored.

## Supporting information

S1 Checklist The ARRIVE guidelines 2.0: Author checklist. (PDF)

S1 Table Panel of genes analyzed by RT-qPCR to identify potential alterations induced by the target miRNA overexpression. (DOCX)

S2 Table AngiomiRs assessed by RT-qPCR in modified MSCs and MSC-EVs. (DOCX)

S3 Table Primers used to assess HUVEC gene expression. (DOCX)
