# Supplementary material for: Overexpression of mir-135b and mir-210 in mesenchymal stromal cells for the enrichment of extracellular vesicles with angiogenic factors
Source: PLoS One. 2022 Aug 16;17(8):e0272962. doi: 10.1371/journal.pone.0272962 (PMC9380919; doi:10.1371/journal.pone.0272962)
Supplement: S1 Table — (DOCX) [file pone.0272962.s002.docx]

**S1 Table**

Panel of genes analyzed by RT-qPCR to identify potential alterations induced by the target miRNA overexpression

| **position** | **unigene** | **GenBank** | **Symbol** | **Description** |
| --- | --- | --- | --- | --- |
| 1 | Mm.277735 | NM 007742 | Col1a1 | Collagen, type I, alpha 1 |
| 2 | Mm.249555 | NM 009930 | Col3a1 | Collagen, type III, alpha 1 |
| 3 | Mm.248380 | NM 011577 | Tgfb1 | Transforming growth factor, beta 1 |
| 4 | Mm.197552 | NM 009370 | Tgfbr1 | Transforming growth factor, beta receptor I |
| 5 | Mm.172346 | NM 009371 | Tgfbr2 | Transforming growth factor, beta receptor II |
| 6 | Mm.282184 | NM 009505 | Vegfa | Vascular endothelial growth factor A |
| 7 | Mm.389712 | NM 010228 | Flt1 | FMS-like tyrosine kinase 1 |
| 8 | Mm.285 | NM 010612 | Kdr | Kinase Insert domain protein receptor |
| 9 | Mm.241282 | NM 010197 | Fgf1 | Fibroblast growth factor 1 |
| 10 | Mm.267078 | NM 010427 | Hgf | Hepatocyte growth factor |
| 11 | Mm.3879 | NM 010431 | HIF1a | Hypoxia inducible factor 1, alpha subunit |
| 12 | Mm.4922 | NM 009969 | Csf2 | Colony stimulating factor 2 (granulocyte-macrophage) |
| 13 | Mm.287228 | NM 009970 | Csf2ra | Colony stimulating factor 2 receptor, alpha, low-affinity (granulocyte -macrophage) |
| 14 | Mm.235324 | NM 007780 | Csf2rb | Colony stimulating factor 2 receptor, beta, low-affinity (granulocyte-macrophage) |
| 15 | Mm.1371 | NM 008781 | Pax3 | Paired box gene 3 |
| 16 | Mm.218760 | NM 011039 | Pax7 | Paired box gene 7 |
| 17 | Mm.3514 | NM 010834 | Mstn | Myostatin |
| 18 | Mm.1526 | NM 010866 | Myod1 | Myogenic differentiation 1 |
| 19 | Mm.4913 | NM 008046 | Fst | Follistatin |
| 20 | Mm.21158 | NM 020009 | Mtor | Mechanistic target of rapamycin (serine/threonine kinase) |
| 21 | Mm.328431 | NM 007393 | Actb | Actin, beta |
| 22 | Mm.343110 | NM 008084 | Gapdh | Glyceraldehyde-3-phosphate dehydrogenase |
| 23 | N/A | SA 00106 | MGDC | Mouse Genomic DNA Contamination |
| 24 | N/A | SA 00104 | RTC | Reverse Transcription Control |
