# Supplementary material for: Overexpression of mir-135b and mir-210 in mesenchymal stromal cells for the enrichment of extracellular vesicles with angiogenic factors
Source: PLoS One. 2022 Aug 16;17(8):e0272962. doi: 10.1371/journal.pone.0272962 (PMC9380919; doi:10.1371/journal.pone.0272962)
Supplement: S2 Table — (DOCX) [file pone.0272962.s003.docx]

**S2 Table**

AngiomiRs assessed by RT-qPCR in modified MSCs and MSC-EVs

| **#** | **Gene Symbol** | **Assay Catalog #** |
| --- | --- | --- |
| 1 | Mm_miR-15a_1 | MS00001281 |
| 2 | Mm_miR-21_2 | MS00011487 |
| 3 | Mm_miR-34a_1 | MS00001428 |
| 4 | Mm_miR-126-3p_1 | MS00005999 |
| 5 | Mm_miR-130a_1 | MS00001547 |
| 6 | Mm_miR-135b_1 | MS00001575 |
| 7 | Mm_miR-135b*_1 | MS00024178 |
| 8 | Mm_miR-210*_1 | MS00024556 |
| 9 | Mm_miR-210_2 | MS00032564 |
| 10 | Mm_miR-221_2 | MS00032585 |
| 11 | Mm_miR-222_2 | MS00007959 |
| 12 | Mm_miR-296-5p_1 | MS00016436 |
| 13 | RNU6-2_11 | MS00032564 |
