# Supplementary material for: Overexpression of mir-135b and mir-210 in mesenchymal stromal cells for the enrichment of extracellular vesicles with angiogenic factors
Source: PLoS One. 2022 Aug 16;17(8):e0272962. doi: 10.1371/journal.pone.0272962 (PMC9380919; doi:10.1371/journal.pone.0272962)
Supplement: S3 Table — (DOCX) [file pone.0272962.s004.docx]

**S3 Table**

Primers used to assess HUVEC gene expression

| Gene | Gene name | NCBI # | Primer | |
| --- | --- | --- | --- | --- |
| *ACTB* | beta-actin | NM_001101.5 | For | GGACCTGACTGACTACCTCAT |
|  |  |  | Rev | CGTAGCACAGCTTCTCCTTAAT |
| *GAPDH* | glyceraldehyde-3-phosphate dehydrogenase | NM_001256799.3 | For | CAAGAGCACAAGAGGAAGAGAG |
|  |  |  | Rev | CTACATGGCAACTGTGAGGAG |
| *HIF 1A* | **hypoxia-inducible factor** 1-alph | NM_001243084.2 | For | GTCTGCAACATGGAAGGTATTG |
|  |  |  | Rev | GCAGGTCATAGGTGGTTTCT |
| *VEGF* | vascular endothelial growth factor | NM_001025366.3 | For | GCTTACTCTCACCTGCTTCTG |
|  |  |  | Rev | CTGTCATGGGCTGCTTCTT |
| *ET-1* | **endothelin-1** | NM_001168319.2 | For | GCAGAAACACACAGTCACATTC |
|  |  |  | Rev | CCTTAGGACCTTCGTCAGAAAC |
| *PGF* | placental growth factor | NM_001207012.1 | For | CAACTGTTTCCCTGCTGAATG |
|  |  |  | Rev | CTGGCTTCTCTCTTTCTCTCAC |
| *INHBA* | **activin A** | NM_002192.4 | For | GTCCTTCCACTCAACAGTCATC |
|  |  |  | Rev | GTACAACATGGACATGGGTCTC |
| *IGFBP1* | insulin-like**growth factor-binding protein** | NM_000596.4 | For | GTCGTAGAGAGTTTAGCCAAGG |
|  |  |  | Rev | ATCCATTCTTGTTGCAGTTTGG |
